# Cellular Mechanism Underlying Formaldehyde-Stimulated Cl^−^ Secretion in Rat Airway Epithelium

**DOI:** 10.1371/journal.pone.0054494

**Published:** 2013-01-23

**Authors:** Yu-Li Luo, Hong-Mei Guo, Yi-Lin Zhang, Peng-Xiao Chen, Yun-Xin Zhu, Jie-Hong Huang, Wen-Liang Zhou

**Affiliations:** School of Life Sciences, Sun Yat-sen University, Guangzhou, China; The Chinese University of Hong Kong, Hong Kong

## Abstract

**Background:**

Recent studies suggest that formaldehyde (FA) could be synthesized endogeneously and transient receptor potential (TRP) channel might be the sensor of FA. However, the physiological significance is still unclear.

**Methodology/Principal Findings:**

The present study investigated the FA induced epithelial Cl^-^ secretion by activation of TRPV-1 channel located in the nerve ending fiber. Exogenously applied FA induced an increase of *I*_SC_ in intact rat trachea tissue but not in the primary cultured epithelial cells. Western blot and immunofluorescence analysis identified TRPV-1 expression in rat tracheal nerve ending. Capsazepine (CAZ), a TRPV-1 specific antagonist significantly blocked the *I*_SC_ induced by FA. The TRPV-1 agonist capsaicin (Cap) induced an increase of *I*_SC_, which was similar to the *I*_SC_ induced by FA. L-703606, an NK-1 specific inhibitor and propranolol, an adrenalin β receptor inhibitor significantly abolished the *I*_SC_ induced by FA or Cap. In the ion substitute analysis, FA could not induce *I*_SC_ in the absence of extracelluar Cl^-^. The *I*_SC_ induced by FA could be blocked by the non-specific Cl^-^ channel inhibitor DPC and the CFTR specific inhibitor CFTR_i-172_, but not by the Ca^2+^-activated Cl^-^ channel inhibitor DIDS. Furthermore, both forskolin, an agonist of adenylate cyclase (AC) and MDL-12330A, an antagonist of AC could block FA-induced *I*_SC_.

**Conclusion:**

Our results suggest that FA-induced epithelial *I*_SC_ response is mediated by nerve, involving the activation of TRPV-1 and release of adrenalin as well as substance P.

## Introduction

For a long time, formaldehyde has been considered as a strongly stimulating environmental pollutant. Prolonged exposure to formaldehyde can cause asthma [1], [2], upper respiratory tract inflammation [3], [4], and pneumonedema, etc. Furthermore, formaldehyde has strong carcinogenic effects on human [5]. In the past, formaldehyde was classified mostly as a compound from extraneous contamination [6]. But recent investigations showed that *in vivo* methylamine can be catalyzed into formaldehyde by semicarbazide-sensitive amine oxidase [7], [8]. The physiological function of this endogenous formaldehyde is still unclear. Studies have demonstrated that endogenous formaldehyde could be related to inflammation responses [9].

TRP (transient receptor potential) channels are a group of non-selective cation channels that consist of six trans-membrane domains. TRP channels can be activated by a variety of factors such as temperature, ligands, mechanical force, pH, etc [10], [11]. They participate in related physiological responses as receptors for mechanical stimuli and other stimulation factors. As a member of the TRP family, TRPV-1 is expressed mostly in the terminals of sensory neurons and it is a mediator sensible to noxious thermal and chemical agents [12], [13]. Studies have demonstrated that TRPV-1 plays an important role in the epidemic inflammation stimulated by formaldehyde [14]. Moreover, the endogenous formaldehyde of tumor tissues can activate TRPV-1 in acidic environment and induce bone cancer pain reactions [15]. Therefore, we hypothesized that formaldehyde might activate TRPV-1 in tracheal nerve endings and trigger a subsequent signal pathway in tracheal epithelium. In order to investigate this idea, we identified TRPV-1 expression in trachea and then we measured *I*_SC_ response in tracheal epithelium in the absence and presence of a variety of pharmacological modulators. According to our results, we propose a model for nerve-dependent regulation of epithelium Cl^-^ secretion in response to formaldehyde stimulation in rat trachea.

## Results

### FA-induced *I*_SC_ Response

**Figure 1:**
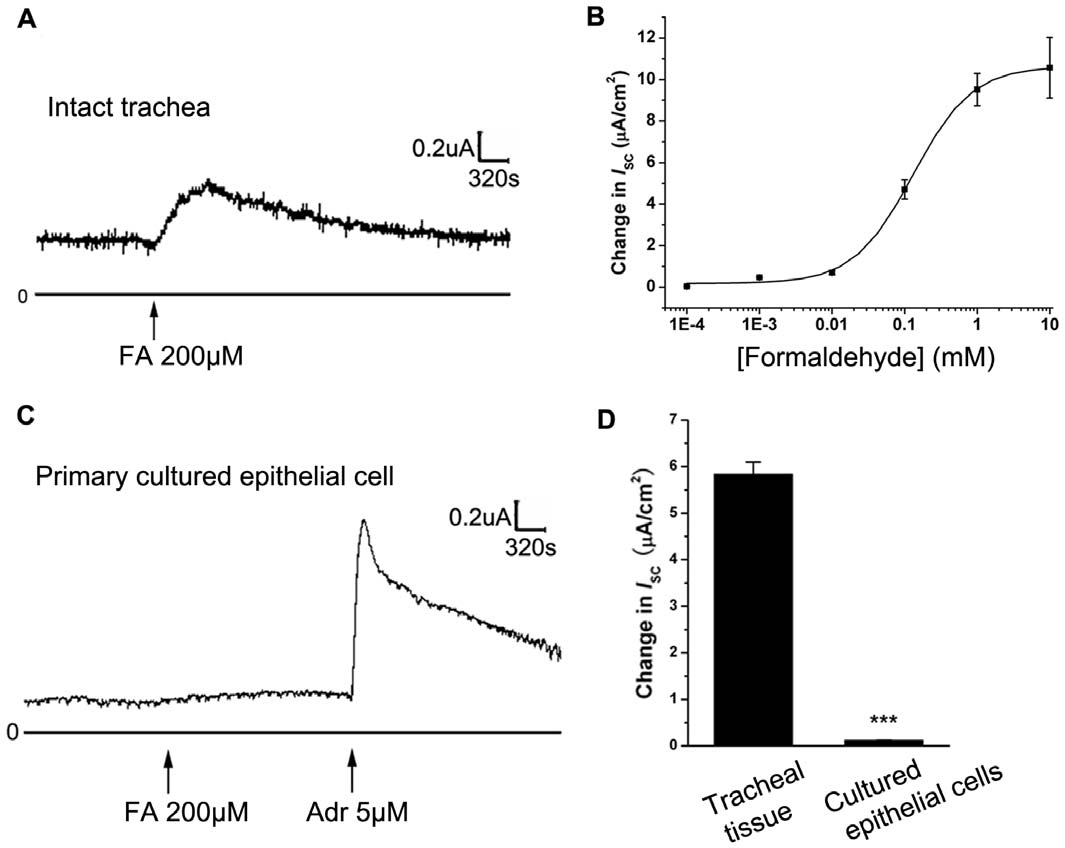
Effect of FA on *I*_SC_ in rat tracheal epithelium. (A) FA 200 µM applied to the basolateral side resulted in an increase in *I*_SC_ in intact tracheal tissue. (B) FA (0.1 µM-10 mM) stimulated a concentration-dependent *I*_SC_ response(n = 5). Each data point represents a mean ± SEM (n = 3∼6). (C) FA (200 µM) applied to the basolateral side resulted no increase in *I*_SC_ in cultured tracheal epithelial cells. Adr (5 µM) was added to demonstrate the good activity of epithelial cells. (D) Comparison of the peak magnitude of *I*_SC_ induced by FA in tracheal tissue and cultured cells. Values are mean ± SEM (n = 4, ***p<0.001).

The basal *I*_SC_ in isolated trachea was 8.22±1.64 µA/cm^2^ (n = 28) and application of FA (200 µM) to the basolateral side caused a sustained increase in *I*_SC_ (Fig. 1A). In addition, FA-induced *I*_SC_ was concentration-dependent (Fig. 1B) with an apparent EC_50_ of about 0.13±0.01 mM. Interestingly, in the primary cultured epithelial cells, FA (200 µM) could not induce an increase in *I*_SC_ (Fig. 1C and D), implying that *I*_SC_ induced by FA was nerve-mediated.

### Expression and Localization of TRPV-1 in Rat Trachea

**Figure 2:**
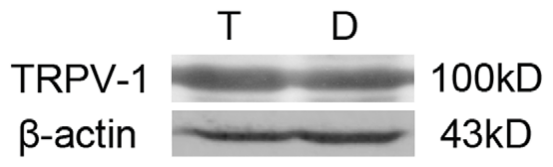
Representative western blot analysis for TRPV-1. T: tracheal tissue; D: dorsal root ganglion (DRG). β-actin served as loading control.

**Figure 3:**
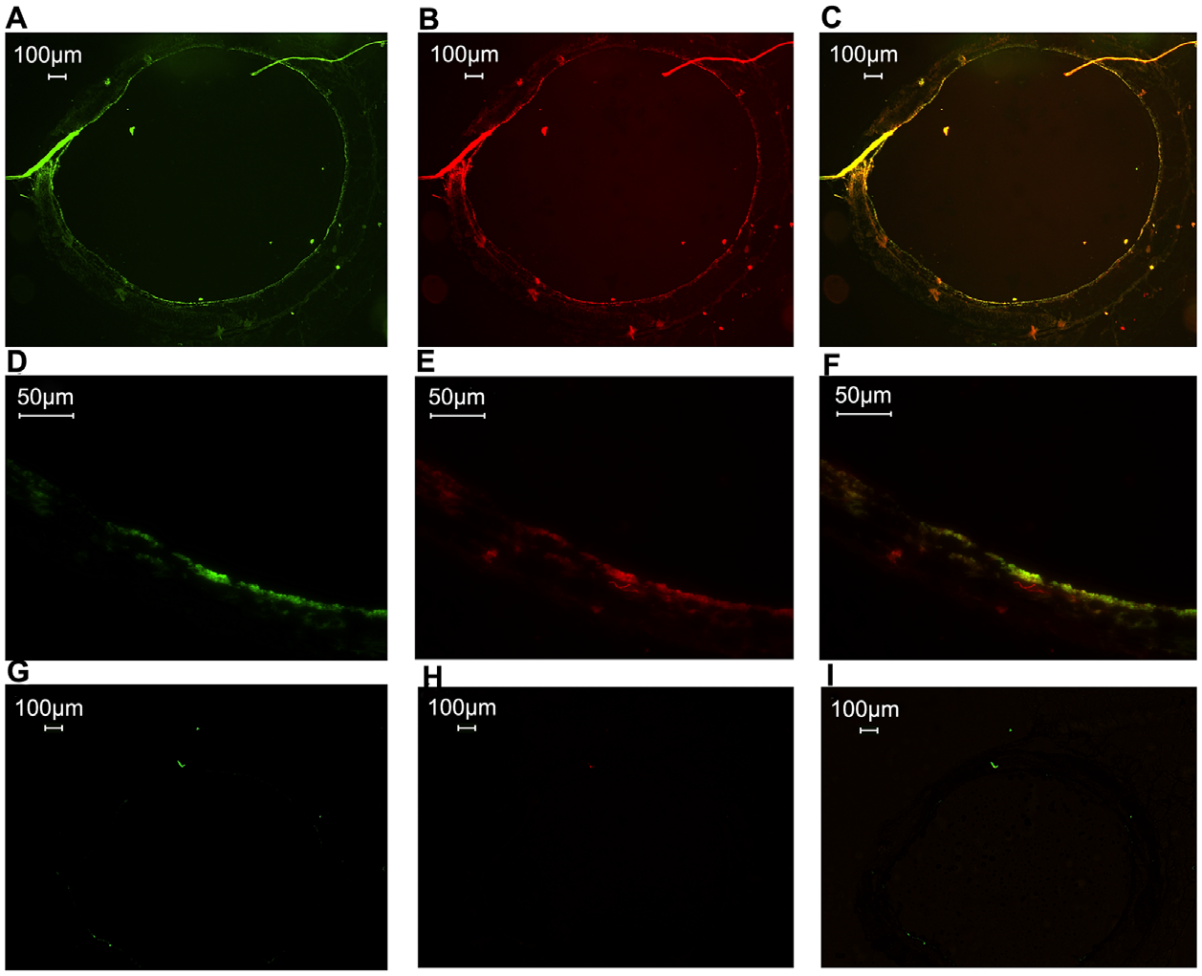
Double immunofluorescence localization of TRPV-1 and neurofilament in rat trachea. (A) and (D) fluorescence images of tracheal sections showing FITC immunoreactivity for TRPV-1. (B) and (E) fluorescence images of tracheal sections showing TR immunoreactivity for NF-H. (C) overlapping of (A) and (B). (F) overlapping of (D) and (E). (G) (H) and (I) negative control, no first antibody was used.

As demonstrated by western blot studies, TRPV-1 was expressed as proteins (Fig. 2). Immunofluorescence was used to clarify the location of TRPV-1. Double labeling TRPV-1 and Neurofilament-H (NF-H), a marker of nerve fibers in trachea showed that the locations of TRPV-1 and NF-H were mostly overlapped (Fig. 3A to F), suggesting that TRPV-1 was largely expressed in the intraepithelial nerve endings. Negative control was directly labeled with secondary antibodies without first antibodies (Fig. 3G, H and I).

### FA-induced *I*_SC_ was Mediated by TRPV-1 in Tracheal Nerve Ending

**Figure 4:**
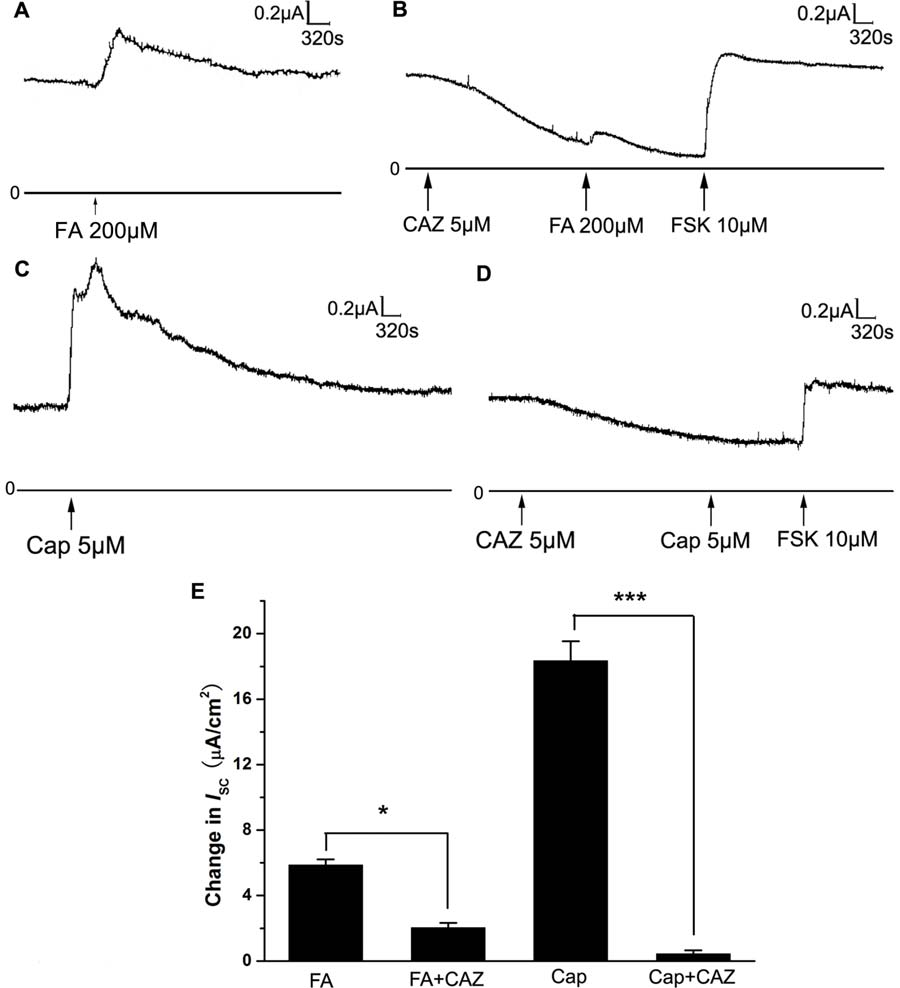
Effects of TRPV-1 agonist and antagonist on *I*_SC_ induced by FA. (A) *I*_SC_ response induced by FA (200 µM). (B) Representative mechanograms show the inhibitory effect of exogenously applied TRPV-1 antagonist CAZ (5 µM) on *I*_SC_ induced by FA (200 µM). (C) Representative mechanograms show the effect of TRPV-1 agonist Cap (5 µM) applied to the basolateral side on *I*_SC_. (D) Representative mechanograms show the inhibitory effect of CAZ (5 µM) applied to the basolateral side on *I*_SC_ induced by Cap (5 µM). FSK (10 µM) applied to the basolateral side was added to demonstrate the good activity of tracheal tissue. (E) Summary results showing the effects of TRPV-1 agonist and antagonist on *I*_SC_ induced by FA (n = 5, mean ± SEM,* p<0.05, *** p<0.001).

In order to investigate the involvement of TRPV-1 in tracheal nerve ending in the *I*_SC_ induced by FA, TRPV-1 specific antagonist and agonist were used. Pretreatment with TRPV-1 specific antagonist capsazepine (CAZ, 5 µM) to the basolateral side of the trachea significantly reduced the subsequent *I*_SC_ induced by FA (Fig. 4B). On the other hand, TRPV-1 agonist capsaicin (Cap, 5 µM) applied to the basolateral side could induce a sustained increase in *I*_SC_ (Fig. 4C), which was similar to the *I*_SC_ induced by FA (200 µM, Fig. 4A), and the Cap-induced *I*_SC_ could be completely abolished by CAZ (5 µM, Fig. 4D). These results clearly demonstrated that TRPV-1 in tracheal nerve ending was involved in the FA-induced *I*_SC_.

### Activation of TRPV-1 Induced the Release of Adrenalin and Activation of β-adrenergic Receptor in Epithelium

**Figure 5:**
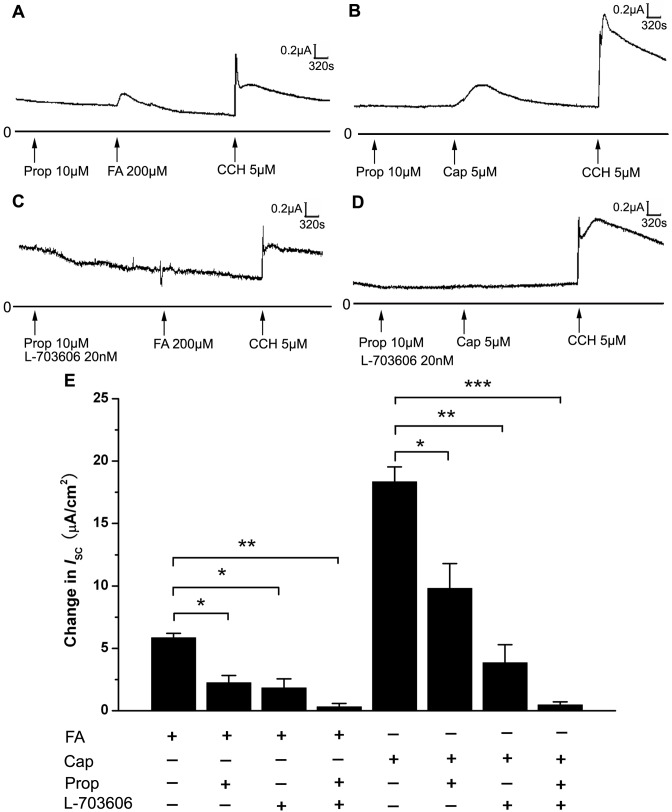
Effect of β-adrenergic receptor and neurokinin-1 receptor on *I*_SC_ induced by FA or **Cap.** Representative mechanograms show the inhibitory effects of exogenously applied the adrenalin β-receptor inhibitor propranolol (Prop, 10 µM) to the basolateral side on *I*_SC_ induced by (A) FA (200 µM) or (B) Cap (5 µM) and exogenously applied Prop (10 µM) as well as the neurokinin-1 (NK-1) specific inhibitor L-703606 (20 nM) to the basolateral side on *I*_SC_ induced by (C) FA (200 µM) or (D) Cap (5 µM). CCH (5 µM) was added to the basolateral side to demonstrate the good activity of tracheal tissue. (E) Summary results showing the effects of Prop (10 µM) and L-703606 (20 nM) on *I*_SC_ induced by FA (200 µM, n = 5) or Cap (5 µM, n = 4, mean ± SEM,* p<0.05, ** p<0.01, *** p<0.001).

The nerve-dependent FA-induced *I*_SC_ might be mediated by the release of neurotransmitters from nerve. It has been reported that activation of TRPV-1 could increase the release of adrenalin [16]. Pretreated the trachea with propranolol (Prop, 10 µM), a β-adrenergic receptor inhibitor to the basolateral side, largely reduced the *I*_SC_ induced by FA (200 µM, Fig. 5A) or Cap (5 µM, Fig. 5B). But the basolateral application of phentolamine (Phen, 10 µM), an α-adrenergic receptor inhibitor, had no effect on the FA-induced *I*_SC_ (Fig. S1). These data indicate that activation of TRPV-1 by FA could release adrenalin from nerve and activate the β-adrenergic receptorβ-adrenergic receptor in epithelium.

### Activation of TRPV-1 also Induced Release of Substance P and Activation of Neurokinin-1 Receptor in Epithelium

Since the β-adrenergic receptor inhibitor Prop could not completely abolish the *I*_SC_ response induced by FA or Cap, it is possible that some other neurotransmitters released from the nerve ending on account of activation of TRPV-1 may be involved in this process. It is well established that activation of TRPV-1 leads to release of substance P. To further test this idea, the neurokinin-1 receptor (NK-1R) specific inhibitor L-703606 (20 nM) was applied to the bathing solution of the basolateral side. The *I*_SC_ induced by FA (200 µM) or Cap (5 µM) was significantly reduced in the presence of L-703606 (Fig. 5E). Furthermore, after pretreatment with both Prop (10 µM) and L-703606 (20 nM), the *I*_SC_ induced by FA (200 µM, Fig. 5C) or Cap (5 µM, Fig. 5D) was almost completely blocked. These results suggest that activation of TRPV-1 may also lead to release of substance P and activation of NK-1R.

### FA-induced *I*_SC_ Response was Mainly Cl^−^ Secretion

**Figure 6:**
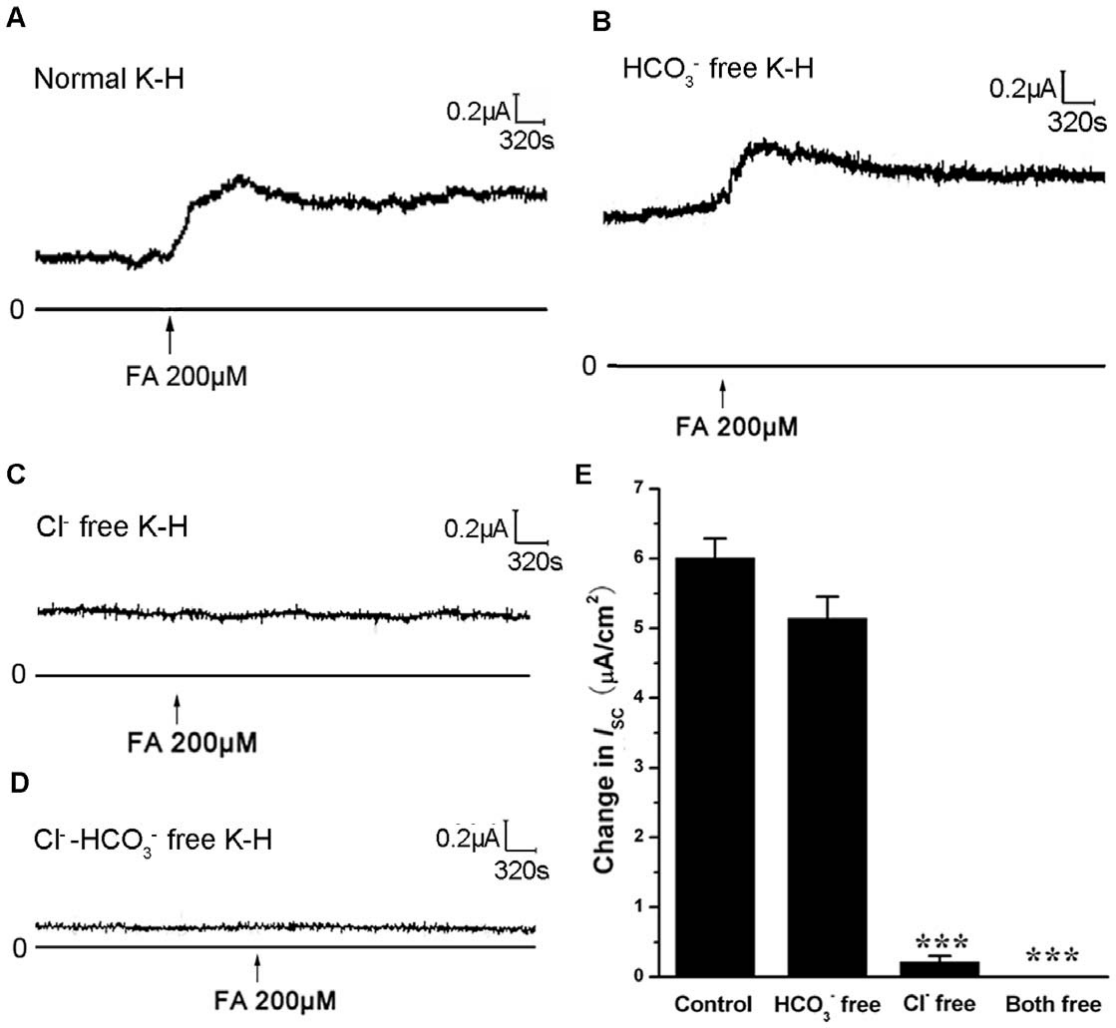
Replacement of extracellular anion resulted in different *I*_SC_ induced by FA. Representative *I*_SC_ recordings with arrows indicating FA (200 µM) were added in different K-H solutions. (A) Normal K-H (B) HCO_3_^−^ free K-H (C) Cl^-^ free K-H and (D) Cl^-^ and HCO_3_^−^ both free K-H. (E) Comparison of the peak magnitude of *I*_SC_ in different bathing solutions induced by FA(200 µM). Values are mean ± SEM (n = 5, ***p<0.001 vs. control).

In order to study the ion species involved in the FA-induced *I*_SC_, a series of ion substitution experiments were conducted. When trachea was bathed in normal K-H solution, FA induced a sustained increase in *I*_SC_ (Fig. 6A). Removing ambient HCO_3_^−^ (HEPES buffering) had little effect on the FA-induced *I*_SC_ response (Fig. 6B). However, removing ambient Cl^-^ (gluconate substitution) or all anionic constituents abolished the FA-induced *I*_SC_ response (Fig. 6C and D). Summary of these effects is shown in Fig. 6E. In addition, application of the epithelial sodium channel (ENaC) inhibitor amiloride (100 µM) to the mucosal side had no effect on the FA-induced *I*_SC_ (Fig. S2), excluding the involvement of Na^+^ reabsorption. All these results suggest that the FA-induced *I*_SC_ is primarily a Cl^-^ current.

### Involvement of cAMP and CFTR in Mediating the Effect of FA

**Figure 7:**
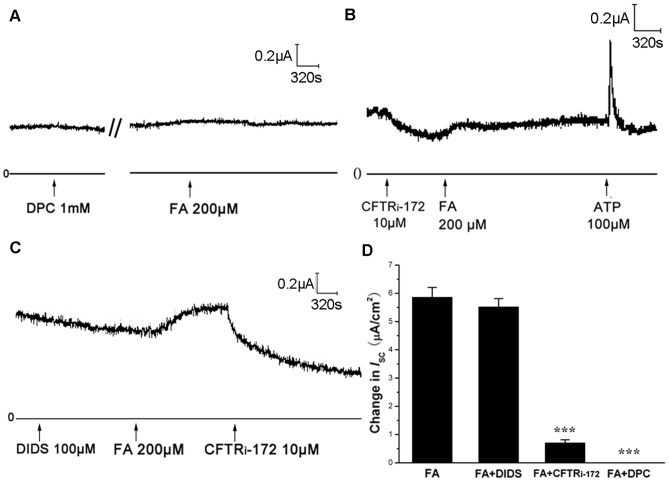
Effect of different chloride channel inhibitors on *I*_SC_ induced by FA. Representative traces showing the ISC induced by FA (200 µM) pretreated with (A) the non-specific chloride channel inhibitor DPC (1 mM) to the mucosal side (B) the CFTR specific inhibitor CFTR_i-172_ (10 µM) to the mucosal side and (C) the Ca^2+^-activated chloride channel (CaCC) inhibitor DIDS (100 µM) to the mucosal side. (D) Summary results showing the effects of different chloride channel inhibitor on *I*_SC_ induced by FA (200 µM). Values are mean ± SEM (n = 6, ***p<0.001 vs. control).

To further figure out which chloride channel participated in FA-induced *I*_SC_ response, different chloride channel inhibitors were applied. Either the non-selective chloride channel inhibitor DPC (1 mM, Fig. 7A) or the CFTR specific inhibitor CFTR_i-172_ (10 µM, Fig. 7B and C) applied to the mucosal side significantly blocked the *I*_SC_ induced by FA. But the Ca^2+^ activated chloride channel (CaCC) inhibitor DIDS (100 µM) applied to the mucosal side had little effect on FA-induced *I*_SC_ (Fig. 7C). Fig. 7D showed the statistical analysis and the comparison of results between different chloride channel inhibitors on the FA-induced *I*_SC_, revealing that the FA-induced Cl^-^ current is mediated by CFTR.

**Figure 8:**
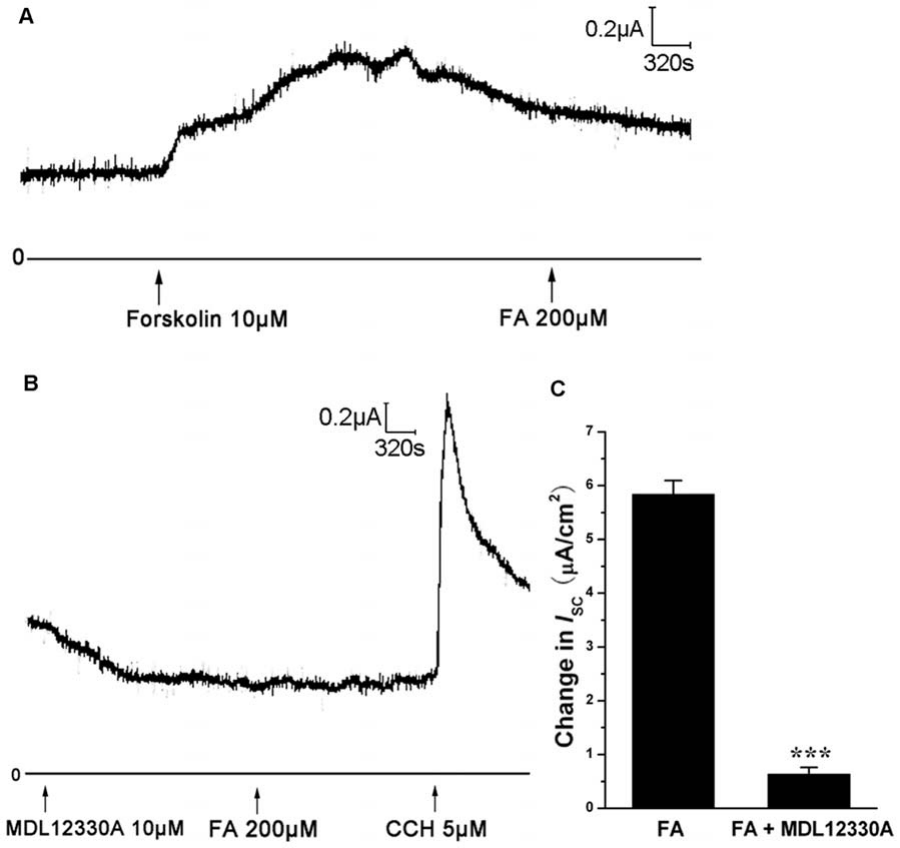
Effect of adenylate cyclase (AC) agonist or antagonist on *I*_SC_ induced by FA. (A) Pretreatment with the AC agonist forskolin (10 µM) to the basolateral side abolished FA-induced *I*_SC_. (B) Pretreatment with the AC antagonist MDL-12330A (10 µM) to the basolateral side decreased FA-induced *I*_SC_. CCH (5 µM) was added to demonstrate the good activity of tracheal tissue. (C) Comparison of the peak magnitude of *I*_SC_ induced by FA with or without MDL-12330A. Values are mean ± SEM (n = 4, ***p<0.001).

Nevertheless, how activation of β-adrenergic receptor could open CFTR and induce Cl^-^ secretion? One possibility is that activation of β-adrenergic receptor might increase the intracellular cAMP level [17] and thus activated CFTR. In order to test this hypothesis, AC agonist and antagonist were applied to the bathing solution. In the presence of forskolin (FSK, 10 µM, an AC activator), which was used to exhaust the AC so that there would be no further elevation in intracellular cAMP, the FA induced *I*_SC_ increase was almost completely abolished (Fig. 8A). Coordinately, pretreatment with MDL-12330A (10 µM, an inhibitor of AC) to the basolateral side of the trachea dramatically reduced the FA-induced *I*_SC_ compared with that in control group (Fig. 8B and C). These results confirm that the FA-induced *I*_SC_ is cAMP dependent.

## Discussion

Formaldehyde had historically been known as a toxic gaseous molecule in the environment. Recent evidences have indicated that FA exists in most cells in human and other animals [18]. The physiological FA level was reported to be approximately 0.1 mM in the blood or brain of human and non-human animals [19]. Interestingly, some evidences have shown that endogenous FA concentration was significantly increased from the patients with tumor [20]-[22]. FA level was also elevated in lymphocytes in chronic lymphocytic leukemia [23]. However, the physiological function as well as the detailed signal pathway involving FA is still unknown yet.

Our present study has demonstrated for the first time that FA could induce a sustained increase in *I*_SC_ in the basolateral aspect of rat intact trachea (Fig. 1A). In addition, applying FA to the mucosal side has a similar effect as applying it to the basolateral side (data not shown).We suppose that this is because as a small-molecule gas, FA can go across the cells swiftly and induce the Cl^-^ currents. Interestingly, in primary cultured rat tracheal epithelial cells, FA could not induce the *I*_SC_ response (Fig. 1C). Since there were no nerve cells in cultured epithelium, there might be some sensors in the nerve ending that participated in the reception of FA and caused the subsequent *I*_SC_ response.

In order to find out the sensor of FA in the nerve, TRPV-1 specific agonist and antagonist were used since previous evidence had suggested that TRP channel might be involved in the sensation of FA. In our experiments, we demonstrated that TRPV-1 was expressed and located in the nerve ending of rat trachea (Fig. 2 and 3). Furthermore, the TRPV-1 specific inhibitor CAZ could significantly block the *I*_SC_ induced by FA (Fig. 4B) and the TRPV-1 agonist Cap could induce a similar *I*_SC_ response as FA did (Fig. 4C). These results indicated that TRPV-1 was involved in the *I*_SC_ induced by FA. Some recent studies also indicated that FA could induce TRPV-1 dependent current in DRG neurons and CHO cells expressing TRPV-1. [15], [24]. However, another group of researchers found that formalin could directly activate TRPA-1 in HEK-293 transfected with hTRPA1 and rTRPA1 respectively [25]. Since in our results CAZ could not completely abolish the *I*_SC_ induced by FA (Fig. 4B and E), TRPA-1 may also be involved in the sensation of FA. One possibility is that at low concentration FA may activate TRPA-1 while at high concentration FA activates both TRPA-1 and TRPV-1. Whether FA at 200 µM could activate TRPA-1 is still controversial and our results supported that FA at 200 µM could activate TRPV-1 in rat trachea.

It is well established that activation of TRPV-1 would induce the calcium influx and secrete a variety of neurotransmitters [26], [27]. Previous evidences also indicated that SP was released in the activation of TRPV-1 [28]-[30]. Our results further confirmed that activation of TRPV-1 would release SP since the NK1R inhibitor could partly block the *I*_SC_ induced by FA or Cap (Fig. 5E). In addition, the AdrR inhibitor Prop also partly abolished the *I*_SC_ response (Fig. 5A, B and E) and pretreatment with the NK1R inhibitor as well as Prop could almost completely block the *I*_SC_ response induced by FA or Cap (Fig. 5C, D and E), implying that not only SP but also adrenalin would be released from nerve endings in activation of TRPV-1.

How does the release of SP and Adr result in the *I*_SC_ response in epithelium? In previous studies, adrenalin could stimulate cAMP-dependent anion and fluid secretion through beta-adrenalin receptor activation [31]-[34]. In addition, SP could stimulate a CFTR-dependent fluid secretion by airway submucosal glands [35]-[37]. In our present study, by using *I*_SC_ measurement, we demonstrated that FA could induce Cl^-^ secretion by inducing the release of adrenalin and SP, for the reason that FA could not induce the *I*_SC_ response in the Cl^-^ free bathing solution (Fig. 6C and D). Furthermore, non specific chloride channel inhibitor DPC and CFTR specific inhibitor CFTR_i-172_ could significantly abolish the *I*_SC_ response whereas CaCC inhibitor DIDS could not (Fig. 7),These data confirmthat FA-induced chloride secretion is mediated by CFTR. TheAC agonist or antagonist could block FA-induced *I*_SC_ response (Fig. 8), further indicating that FA induces a cAMP-dependent CFTR-mediated chloride secretion.

**Figure 9:**
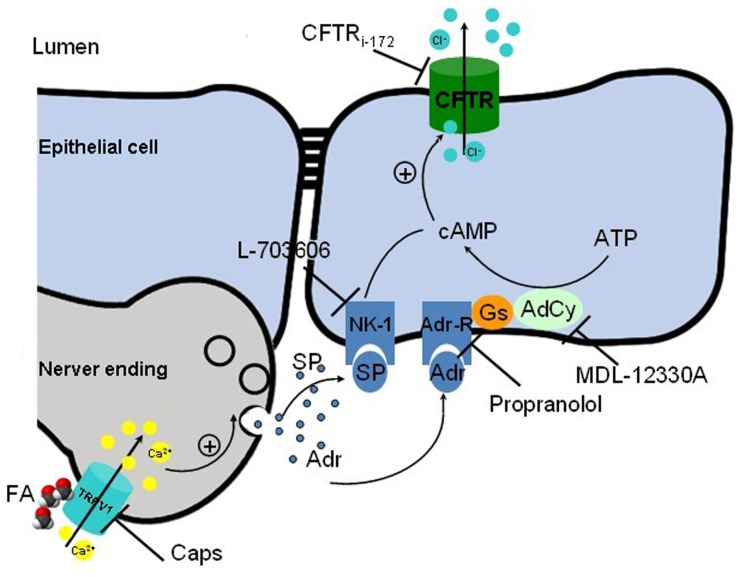
Cellular mechanism of formaldehyde caused Cl^-^ secretion in nerve-dependent epithelium. FA activates TRPV-1 in tracheal nerve ending, causing a Ca^2+^ influx, thus induces the release of SP and Adr in the nerve. SP activates NK1R and Adr activates β-AdrR in epithelium, evoking the increase of cAMP and finally activating CFTR to induce a Cl^-^ secretion in tracheal epithelium.

Based on the present findings, we propose a working model for nerve-epithelium intercellular communication. FA works as an important stimulus and SP as well as Adr are nerve-derived factors that regulate the tracheal epithelium anion secretion (Fig. 9). FA activates TRPV-1 in tracheal nerve ending, causing a Ca^2+^ influx, and thus induces the release of SP and Adr in the nerve. SP activates NK1R and Adr activates β-AdrR in epithelium, evoking the increase of cAMP and finally activating CFTR to induce a Cl^-^ secretion in tracheal epithelium. Interestingly, in addition to their effects on epithelium, nerve-derived factors may also influence smooth muscle cells or immune cells. For example, adrenalin could relax airway smooth muscles through the mediation of β2-adrenoceptors [38]. Clinical studies also suggest that beta-receptor is an important target for the treatment of asthma and COPD [39]–[41]. In our experiments, FA-induced epithelium Cl^-^secretion would further trigger the fluid transportation through epithelium. Therefore, in our model FA induces a nerve-derived release of adrenalin and SP leading to a CFTR-dependent chloride and fluid secretion, which might act as an innate defense response and would regulate the airway surface liquid volume in the extracellular environment. Our model also suggests an SP related inflammatory pathway at the high concentration of FA since SP is a well-known inflammatory factor. On the other hand, SP can activate NK1R in epithelium, evoking COX, which results in the generation of PGE_2_ and activates EP_2/4_ receptors in airway smooth muscle, leading to an increase in cAMP [42]. This study provides a possible pathway in our model that PGE_2_ may also activate EP_2/4_ receptors and increase the level of cAMP in epithelium to activate CFTR. In summary, the present study demonstrates that SP and Adr are important nerve-derived factors in FA-induced nerve-dependent CFTR-mediated Cl^-^ secretion. Thus, the present results provide a new insight into the physiological role of endogenous formaldehyde.

## Materials and Methods

### Ethics Statement

SPF Sprague-Dawley rats of 180 g –200 g were purchased from the Experimental Animal Center of Guangdong Province. Animals were housed in a constant-temperature (25°C) room with a 12 h light–12 h dark photoperiod, according to the guidelines of the Sun Yat-sen University Animal Use Committee. All procedures were approved by the Sun Yat-sen University Animal Use Committee.

### Measurement of *I*_SC_

For all *I*_SC_ measurements, the main trachea of the rat airway system was cut into appropriate size and then mounted in hallowing paraffin between two halves of modified Ussing Chambers with an internal area of 0.03 cm^2^. Electrodes for measuring transepithelial potential difference (PD) and passing current were connected to the chamber. The transepithlial PD was clamped at 0 mV, and then the short circuit currents were recorded with VCC MC6 voltage-current clamp amplifier (Physiologic instrument, San Diego, CA) and simultaneously displayed via a signal collection and analysis system (BL-420E^+^, Chengdu Technology & Market Co. Ltd, China). The change in *I*_SC_ was defined as the maximal rise in *I*_SC_ following agonist stimulation and was normalized to current change per unit area of the epithelial monolayer (µA/cm^2^). The *I*_SC_ value was expressed as positive when the current flow from basolateral to apical [43].

### Western Blotting

Total protein extract was obtained from rat trachea and dorsal root ganglion. The supernatant was collected from tissue homogenate after centrifugation. Equal amount of protein loaded in each lane was resolved by SDS-polyacrylamide gel electrophoresis and blotted on PVDF membrane. Membranes were blocked in Tris-buffered saline (TBS)/0.1% Tween-20 with 5% nonfat milk powder for 30 minutes at room temperature, and then incubated with specific antibody (Santa Cruz) for 2 h at room temperature. Membranes were incubated with peroxidase conjugated second antibody (Wuhan Boster Bio-engineering, Co. Ltd) for 1 h. The labeled proteins were detected with ECL kit.

### Immunofluorescence

The main trachea of the rat airway system was removed rapidly and frozen in liquid nitrogen. Frozen tissues were sectioned by using a freezing microtome (Leica CM1850UV). For double staining, after the sections were pre-incubated with goat serum to minimize non-specific binding of IgG, they were incubated with first antibodies (rabbit TRPV-1, 1∶200; mouse Neurofilament-H, NF-H, 1∶200; Santa Cruz) at 4°C overnight. After washed for 3 times, sections were incubated with the appropriate secondary antibodies (anti-rabbit IgG-FITC, 1∶100; anti-mouse IgG-TR, 1∶100; Santa Cruz) for 1 h, then washed again in PBS, dehydrated in ethanol, air-dried, and mounted in Fluoromount. The specimens were examined under a fluorescence microscope (Leica DM5000B) at 546 nm and 490 nm wavelength.

### Cell Culture

The procedures of tracheal epithelium primary culture have been described previously [44]. In brief, Sprague-Dawley rats weighing 180 g –200 g were killed by CO_2_ inhalation. The main trachea was dissected out, finely minced with scissors, and treated successively with 0.25% (w/v) trypsin and 0.1% (w/v) collagenase. The disaggregated cells were suspended in Dulbecco’s modified Eagle’s medium/F-12 (DMEM/F-12) supplemented with 10% (v/v) fetal bovine serum, 100 U/ml penicillin and 100 mg/ml streptomycin. After primary culture of 4–6 h, the cells were seeded onto Millipore filters (0.45 cm^2^) floating on DEME/F12 completed with other supplements. Cultured cells were incubated for 4 ∼ 5 days at 37°C in 5% CO_2_/95% O_2_. Thereafter, the monolayers reached confluence and were ready for the measurement of *I*_SC_.

### Solutions and Chemicals

The normal KH solution contained (in mM) 117 NaCl, 4.7 KCl, 1.2 MgSO_4_, 1.2 KH_2_PO_4_, 25 NaHCO_3_, 2.56 CaCl_2_, and 11.1 Glucose. The solution was kept at 37°C and gassed with 95% O_2_ and 5% CO_2_ to maintain the pH at 7.4. In Cl^−^ free Kreb’s solution, NaCl, KCl and CaCl_2_ were replaced with the representative salts of gluconate, other compositions and condition were not altered. In HCO_3_^−^ free solution, HCO_3_^−^ was substituted by 10 mM HEPES and the solution were bubbled with 100% O_2_. In Cl^-^ and HCO_3_^−^ free solution, the component is the same as Cl^-^ free solution, except NaHCO_3_ was replaced with 20 mM Na-gluconate and 10 mM HEPES, and in the solution the gas was 100% O_2_.

Dulbecco’s modified Eagle’s medium/F-12 (DMEM/F-12), fetal bovine serum (FBS), penicillin/streptomycin, Hanks balanced salt solution and trypsin were purchased from Gibco Laboratories. DPC was bought from Riedel-de-Haen. Capsaicin, capsazepine, L-703606, propranolol, phentolamine, DIDS, CFTR_i-172_, forskolin, MDL-12330A, CCH were purchased from Sigma Chemical Co. Rabbit TRPV-1, rabbit β-actin, mouse neurofilament-H, anti-rabbit IgG-FITC and anti-mouse IgG-TR were obtained from Santa Cruz biotechnology, INC.

### Data Analysis and Statistics

Data were presented as Mean ± S.E.M. (n is the number of tissue preparations, or cells, or experiment times). For two groups of data, two-tail Student’s t test was used. For more than three groups of data, ANOVA followed by Bonferroni’s post hoc test was used. A value of P<0.05 was considered to be statistically significant.

## Supporting Information

Figure S1 **Effect of adrenalin α-receptor inhibitor on** ***I*** **_SC_ induced by FA.** Representative mechanograms show the exogenously applied adrenalin α-receptor inhibitor phentolamine (Phen, 10 µM) to the basolateral side had no effect on *I*_SC_ induced by FA (200 µM) on tracheal tissue.
(TIF)

Figure S2 **Effect of sodium-hydrogen exchanger inhibitor on** ***I*** **_SC_ induced by FA.** Representative mechanograms show the exogenously applied the epithelial sodium channel (ENaC) inhibitor amiloride (100 µM) to the mucosal side had no effect on *I*_SC_ induced by FA (200 µM) on tracheal tissue.
(TIF)
