# Supplementary figures and images for: Cellular Mechanism Underlying Formaldehyde-Stimulated Cl− Secretion in Rat Airway Epithelium
Source: PLoS One. 2013 Jan 23;8(1):e54494. doi: 10.1371/journal.pone.0054494 (PMC3553115; doi:10.1371/journal.pone.0054494)

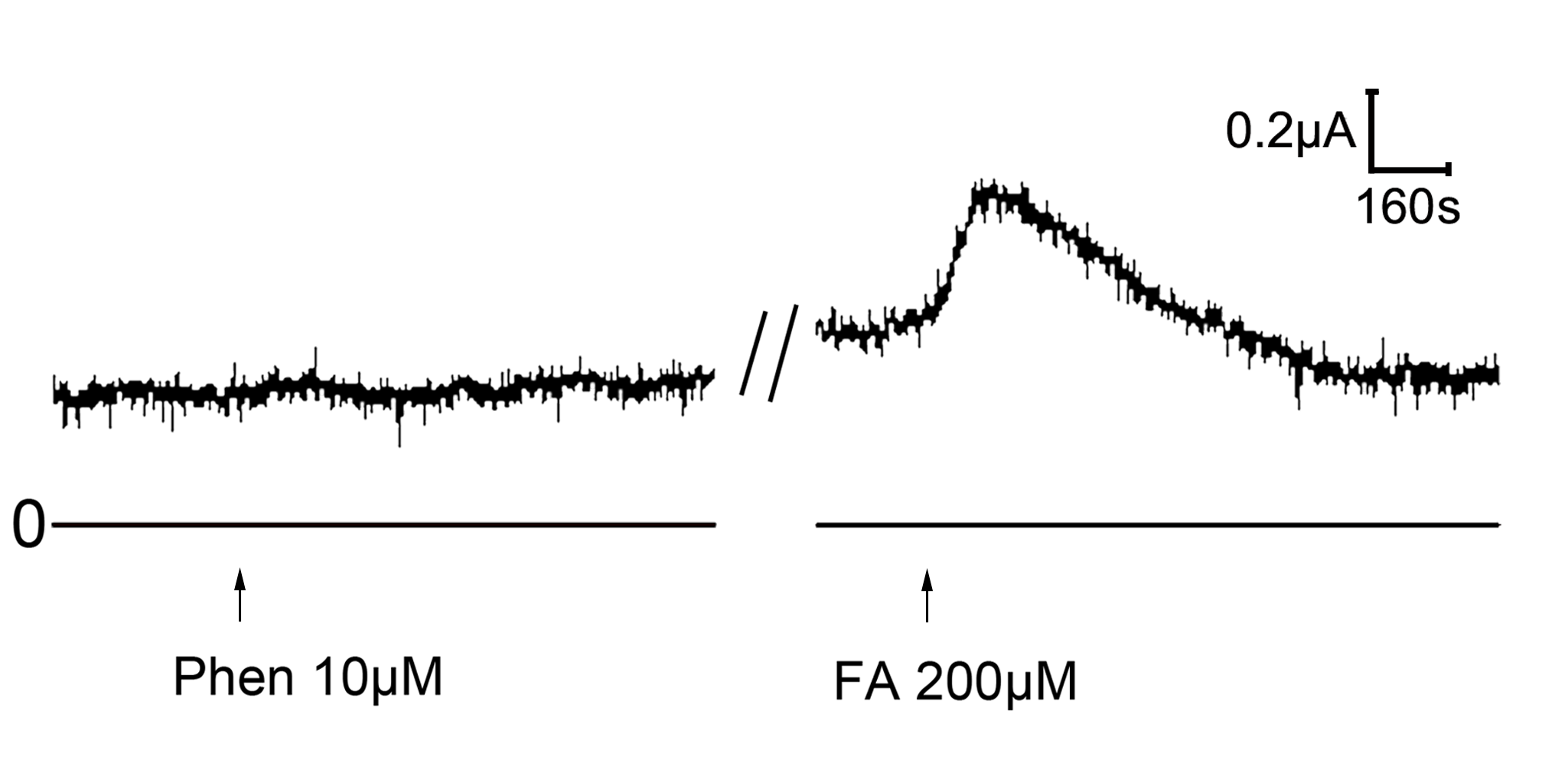

Supplement: Figure S1 — Effect of adrenalin α-receptor inhibitor on I SC induced by FA. Representative mechanograms show the exogenously applied adrenalin α-receptor inhibitor phentolamine (Phen, 10 µM) to the basolateral side had no effect on I SC induced by FA (200 µM) on tracheal tissue. (TIF) [file pone.0054494.s001.tif]

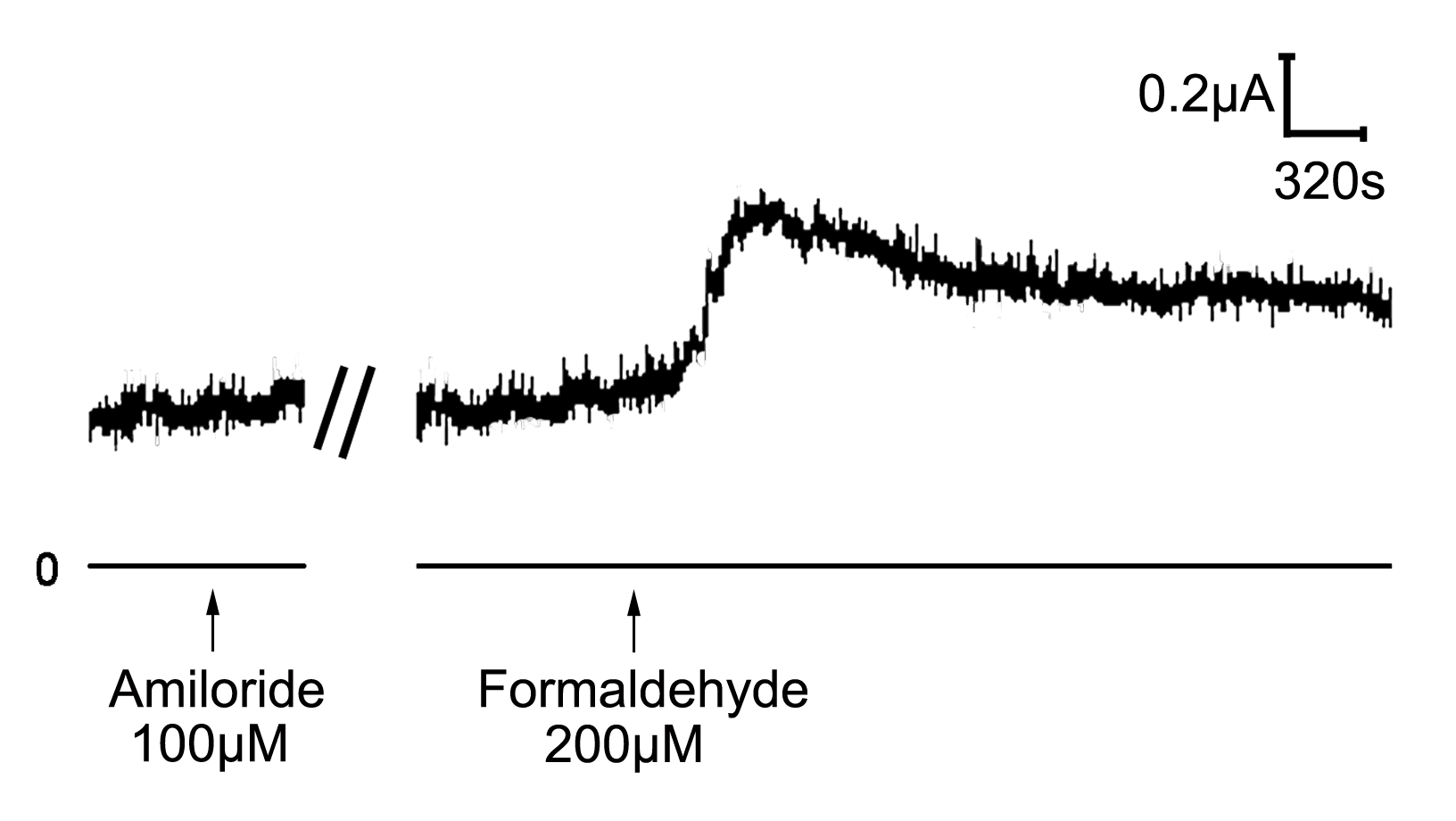

Supplement: Figure S2 — Effect of sodium-hydrogen exchanger inhibitor on I SC induced by FA. Representative mechanograms show the exogenously applied the epithelial sodium channel (ENaC) inhibitor amiloride (100 µM) to the mucosal side had no effect on I SC induced by FA (200 µM) on tracheal tissue. (TIF) [file pone.0054494.s002.tif]
